# Primary Extramedullary, Extradural Cervical Spine Seminoma

**DOI:** 10.5435/JAAOSGlobal-D-19-00177

**Published:** 2020-07-02

**Authors:** Charles Long, Thomas A. Novack, Stuart Changoor, Kumar Sinha, Ki Soo Hwang, Michael J. Faloon, Arash Emami

**Affiliations:** From the Department of Orthopaedic Surgery, St. Joseph's University Medical Center, Paterson, NJ.

## Abstract

While extragonadal seminomas resulting in spinal cord compression are rarely reported in the literature, most have been treated with surgical decompression followed by radiation therapy. In this report, we present the unique and interesting case of a 38-year-old man who initially presented as an outpatient with a chief complaint of axial neck pain and lateral thoracic wall pain. After an extensive malignancy workup, he was diagnosed with a primary cervical spine seminoma and was treated with a C6–T1 laminectomy with posterior spinal instrumentation from C5 to T2. He has since undergone chemotherapy with cisplatin, vinblastine, and bleomycin, and at 24-month follow-up, he remains asymptomatic with no signs of recurrent disease.

Malignant spinal cord compression occurs in 5% to 14% of all patients diagnosed with cancer. This is most commonly caused by metastatic disease from a primary lesion including tumors of the prostate, lung, and breast. Primary germ cell tumors of the central nervous system are uncommon, typically intramedullary, and affect the pineal region. Although extremely rare, there have been several reports of primary germinomas of the spine and therefore they should be considered in the differential diagnosis of a cervical spinal mass in a young patient.^1^ Typical treatment consists of corticosteroids, radiation therapy, and, when possible, resection of the mass.^2,3^ In this report, we describe an unusual case of a patient with a primary extragonadal seminoma, a germ cell tumor, located in the posterior elements of the cervical spine causing malignant spinal cord compression.

## Case Report

### Patient Presentation

**Figure 1:**
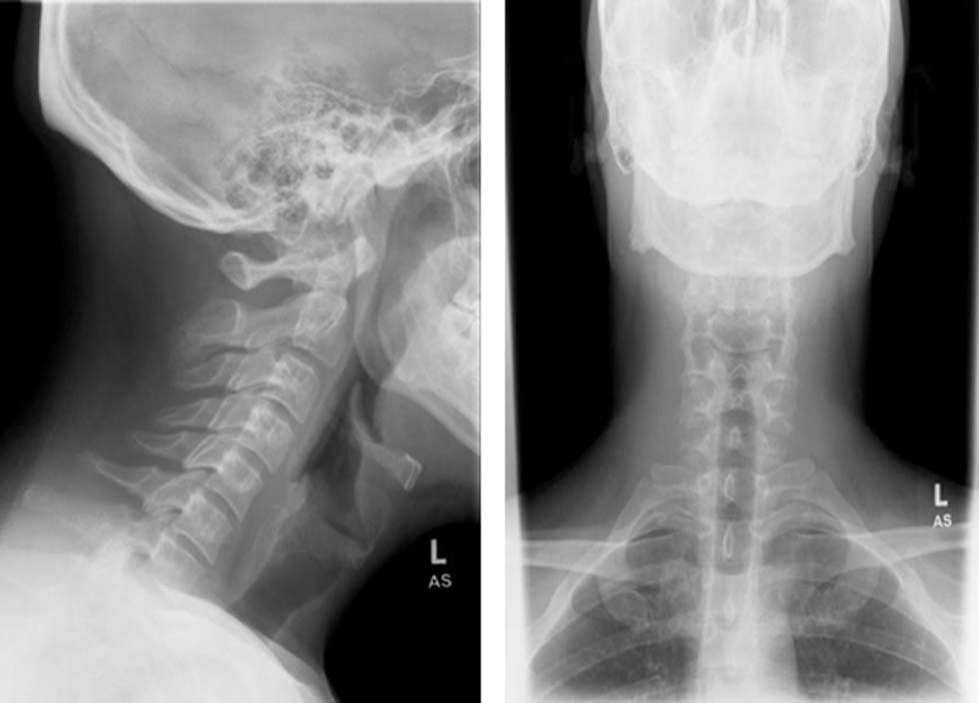
Initial X-rays of the cervical spine (AP and lateral views) demonstrating no obvious spinal pathology.

Our patient is a 38-year-old man with no significant medical history who initially presented to the outpatient clinic with a 1-month history of axial neck pain and right lateral thoracic pain. The pain was localized to the right 11th intercostal space and was exacerbated by inspiration. He reported no trauma or prior inciting event. On physical examination, he had tenderness to palpation about his posterior neck. Motor and sensory exams were within normal limits with no obvious neurologic deficit. Initial plain AP and lateral radiographs of the cervical spine were normal (Figure 1). The patient was initially treated conservatively with NSAIDs and physical therapy. However, 6 weeks later, the patient returned without symptomatic improvement and new onset right upper extremity radiculopathy.

### Diagnostic Imaging and Workup

**Figure 2:**
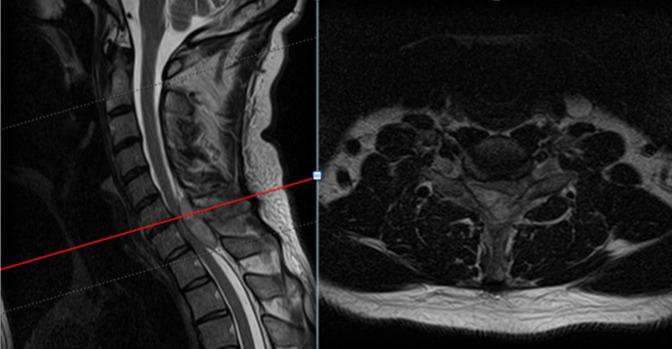
Sagittal and axial MRI images of the cervical spine at the C6–7 level demonstrating the posterior spinal lesion leading to spinal cord compression.

**Figure 3:**
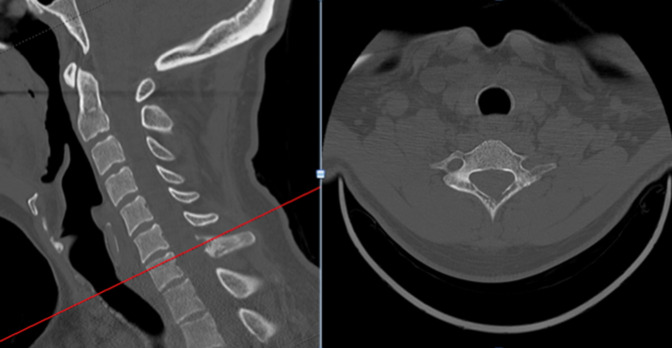
Sagittal and axial CT images of the cervical spine showing erosive changes to the C7 spinous process.

MRI of the cervical spine was obtained and demonstrated a nongeographic, destructive lesion in the posterior elements of the cervical spine arising from the spinous process of C7 and evidence of spinal cord compression (Figure 2). CT scan of the cervical spine demonstrated erosive changes to the C7 spinous process (Figure 3). A malignancy workup was performed, and an oncology consult was obtained. Chest/abdomen/pelvis CT scan revealed metastatic disease of the right 11th rib; however, no primary mass was identified. Bone scan showed increased uptake in the right 11th rib, but no other evidence of metastatic bone disease. No primary malignancy was identified on the initial screening. Given the nature of the patient's symptoms and MRI findings, the patient was indicated for surgical intervention for primary resection of the tumor.

### Surgical Management and Follow-up

**Figure 4:**
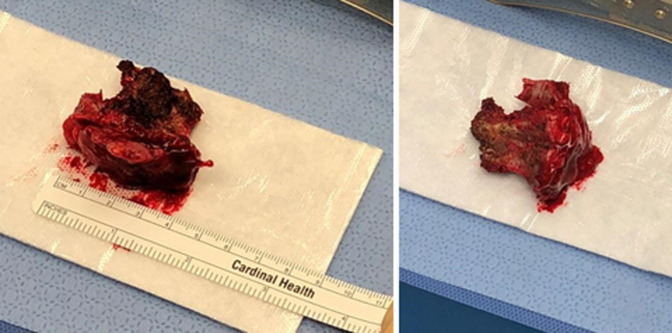
Photographs of the resected specimen containing lamina and tumor.

**Figure 5:**
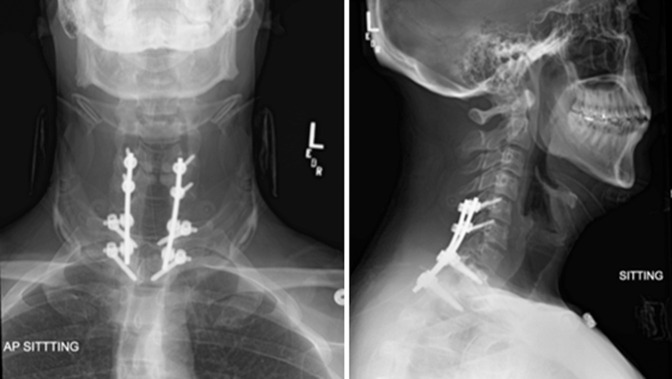
Postoperative AP and lateral cervical spine radiographs demonstrating posterior spinal instrumentation from C5 to T2.

**Figure 6:**
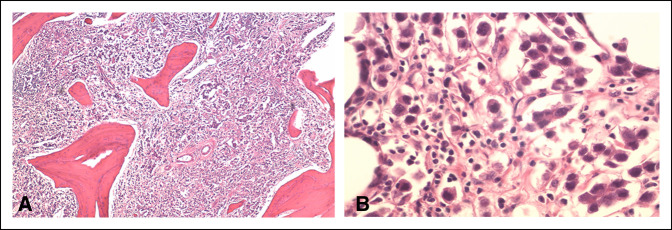
**A**, Low power field histology image demonstrating bony trabeculae containing both tumor cells and lymphocytes. **B**, High power field histology image demonstrating dark small lymphocytes and squared large seminoma cells.

**Figure 7:**
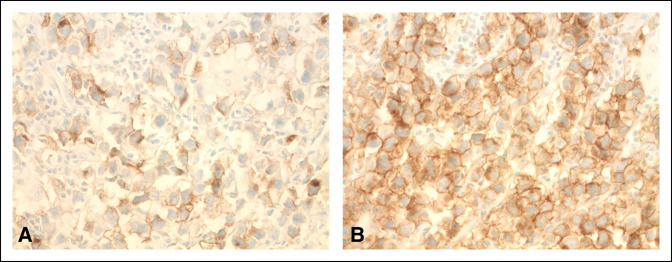
Histology slides with (**A**) placental alkaline phosphatase and (**B**) proto-oncogene c-Kit (CD117) stain demonstrating the presence of seminoma cells.

A C6–T1 laminectomy was performed to gain access to the mass. The tumor was identified and wide en bloc resection of the mass was performed. The specimen consisted of the lesion surrounded by healthy appearing tissue (Figure 4). Frozen section of excised tissue was positive for numerous malignant cells with negative margins. Posterior spinal instrumentation from C5 to T2 was then performed (Figure 5). Histology slides demonstrated bony trabeculae with both, tumor cells and lymphocytes, on low power field (Figure 6, A). Dark small lymphocytes and large square seminoma cells were present on high power field (Figure 6, B). Placental alkaline phosphatase stain (Figure 7, A) and proto-oncogene c-Kit (CD117) stain (Figure 7, B) were positive for seminoma cells. Final pathology was positive for seminoma, confirming this diagnosis. A testicular ultrasonography was performed to investigate for the presence of a primary testicular tumor, but was negative for malignancy. The patient was discharged from the hospital on postoperative day 5 with orthopaedic and oncologic follow-up. He has since recovered well and has undergone three cycles of chemotherapy with cisplatin, vinblastine and bleomycin. Postoperative radiographs show hardware in excellent position. At 24-month follow-up, he was asymptomatic with no evidence of recurrence of the spinal mass or progression of the rib lesion. He continues to be monitored with periodic spine and testes MRIs and chest CT scans.

## Discussion

Seminomas represent 50% of all germ cell tumors and most commonly affect white men between the ages of 20 to 40. There are approximately 4,000 new cases per year in the United States, representing a small fraction of new oncologic diagnoses. The prevalence of these tumors is greater in populations with a history of radiation exposure. Although classically found in the testes, extragonadal lesions have been described. Extragonadal seminomas represent 2% to 5% of all germ cell tumors and primarily arise from structures within the mediastinum, retroperitoneum, and pineal gland. Within the mediastinum, primary extragonadal seminomas have been found to arise from cardiovascular and pulmonary structures.^4,5^

Patients with primary testicular seminomas typically present with a painless testicular mass. At the time of diagnosis, 80% of these patients have disease limited to the testes without evidence of metastasis. The treatment of primary testicular seminomas includes resection and radiation therapy. With orchiectomy and radiation therapy, patients can expect cure rates up to 99% without recurrence.^6^ Most commonly, seminomas metastasize via the lymphatic system to the viscera such as the lungs. Although rare, both metastatic seminomas and primary extragonadal seminomas can involve the spine and result in spinal cord compression.^7^

Four cases of spinal cord compression due to metastasis from a primary testicular seminoma have been previously reported in the literature. Yee et al^7^ reported 2 cases of thoracic spinal metastases. The first describes a 33-year-old man who had previously undergone right inguinal orchiectomy and was under surveillance for recurrence. The patient developed metastases to the spine and suffered a pathologic T12 burst fracture requiring decompression and fusion from T10 to L2. The second case describes a 44-year-old man who presented with back pain and lower extremity weakness. He was found to have metastatic lesions to T4 and T5 with spinal cord compression. After left inguinal orchidectomy, combination cisplatin, etoposide, and bleomycin chemotherapy, external beam radiation therapy, his motor function and sensation improved. He did not require surgical decompression. Ng et al.^8^ reported a third case of spinal cord compression due to recurrent metastatic testicular seminoma. The authors described a case of a 38-year-old man who presented 14 months' status post right inguinal orchiectomy with back pain, lower extremity weakness, and acute urinary retention. He underwent posterior decompression from T7 to T9 and posterior instrumentation from T6 to T11. He regained full neurological function postoperatively. Chiba et al^9^ also reported a case of a patient who developed gait disturbances secondary to metastatic seminoma 6 years after radical orchiectomy. The patient was found to have a new T8 lesion on routine surveillance and experienced full recovery of his symptoms after surgical decompression and fusion.

In patients with primary retroperitoneal and mediastinal seminomas, extensive mass effect and destruction of the anterior spinal elements must occur before spinal cord compression results.^7^ In a case series of 48 patients with primary extragonadal germ cell tumors, 27 arose from the central nervous system, 16 from the mediastinum, and 5 from the retroperitoneum, none of which presented with spinal cord compression.^10^ Another case series of extragonadal manifestations of seminomas in the head and neck area included 16 patients, but none involved the cervical spine or resulted in spinal compression.^11^ There was one case of spinal cord compression due to a primary posterior mediastinal germ cell tumor reported in the literature in a 7-year-old boy. The mass extended from T5 to T11 with involvement of the vertebral bodies of T8–T9. The patient was treated with total laminectomy from T6 to T10 with postoperative bracing treatment. The patient recovered postoperatively with mild kyphotic curvature and 4/5 strength in his lower extremities at 3 years postoperatively.^12^

Primary spinal germinomas have also been described although they typically arise from the medulla of the spinal cord itself. In a literature review by Loya et al,^13^ 30 primary intradural spinal germinomas were reported, only 13% of which were purely extramedullary. All four of the cases of primary extramedullary spinal germinomas were intradural, requiring posterior laminectomy and durotomy to access the lesions, and careful and extensive dissection of the tumor from the nerve roots with variable clinical success (Table 1). Each patient underwent subsequent treatment with cisplatin, etoposide, and bleomycin chemotherapy. The first case described a 28-year-old man who required six laminectomies and resections before achieving remission after 22 months.^14^ A second case describes a 28-year-old man who died secondary to the cardiopulmonary complications of transverse myelitis from ascending metastases to the cervical spine.^5^ Finally, two case reports, one of a 43-year-old man and another of a 20-year-old woman, describe near full neurologic recovery after posterior decompression and dissection of the nerve roots.^9,15^

To the best of our knowledge, this is the first report of a primary extragonadal, extradural seminoma in the posterior elements of the cervical spine. This highlights the importance of the unique location of the tumor, which presents a diagnostic challenge as to the identity of the tumor prior to biopsy. Surgical management is also dependent on the location of the tumor because durotomy was not required to access our patient's tumor due to its extradural nature. Certainly, it is imperative to do a thorough oncologic workup before surgical resection. If a primary seminoma is identified, primary resection followed by an oncological consultation should be highly considered because previous case reports have supported the benefits of surgical decompression and subsequent radiation and chemotherapy.

**Table 1:** Existing Case Reports of Primary Extragonadal Extramedullary Seminomas of the Spine

Author/Year	Age at Diagnosis	Presentation	Spinal Level	Location	Surgical Decompression	Adjuvant Treatment	Outcome	Complications
Tekkök and Sav,^14^ 2005	28M	Back and leg pain, acute urinary incontinence (2 mo)	L1–S2	Intradural	Six multilevel laminectomies, dissection from the nerve roots	BEP chemotherapy	22 mo, no recurrence after final re-resection, paraplegic	Recurrence at multiple levels requiring five re-resections
Biswas et al,^5^ 2009	28M	Back pain, lower extremity weakness (2 mo)	L2–L4	Intradural	L2–L4 laminectomy, durotomy, dissection from the nerve roots	BEP chemotherapy, EBRT (20 Gy)	11 mo, death from cardio-respiratory arrest due to compressive transverse myelitis	Recurrence and ascending metastasis with arachnoiditis at 3 mo, cervical transverse myelitis from metastatic compression, death
Horvath et al,^16^ 2001	43M	Back pain, acute cauda equina (2 mo)	L1–L2	Intradural	T12–L3 laminectomy, durotomy, dissection from the nerve roots	BEP chemotherapy, EBRT (36 Gy)	8 mo, no recurrence, residual sphincter dysfunction	Bleomycin pneumonitis
Kiyuna et al,^15^ 1999	20F	Back pain, lower extremity weakness, acute urinary incontinence (2 yr)	T11–L3	Intradural	T10–L3 laminectomy, durotomy, dissection from the nerve roots	EBRT (40 Gy)	2 yr, no recurrence, asymptomatic	—
Present case, 2019	38M	Neck and right lateral thoracic pain (2 mo)	C7	Extradural	C6–T1 laminectomy, C5–T2 posterior spinal instrumentation	BEP chemotherapy	24 mo, no recurrence, asymptomatic	—

BEP = cisplatin, etoposide, and bleomycin, EBRT = external beam radiation therapy
